# Association Between Childhood Neglect and Major Depressive Disorder: A Systematic Review, Meta-Analysis and Meta-Regression

**DOI:** 10.1192/j.eurpsy.2026.10597

**Published:** 2026-07-31

**Authors:** M. Merola, S. Carletto, L. Ostacoli, F. Oliva

**Affiliations:** 1 Department of Clinical and Biological Sciences; 2 Department of Psychology, University of Turin, Turin, Italy

## Abstract

**Introduction:**

Major depressive disorder (MDD) is one of the most prevalent psychiatric conditions worldwide and is strongly associated with early adverse experiences. While the impact of childhood abuse has been extensively studied, the specific contribution of neglect, encompassing physical, emotional, and unspecified subtypes, has historically received less attention. Given its potential to disrupt attachment and emotion regulation, quantifying its association with MDD is crucial.

**Objectives:**

This study aimed to investigate the association between childhood neglect and MDD by estimating pooled Odds Ratios (ORs) for unspecified, physical, and emotional neglect. A secondary objective was to examine moderators of these associations through meta-regression analyses.

**Methods:**

Following PRISMA guidelines, PubMed, Scopus, and Web of Science were systematically searched in September 2024. Eligible studies included adults with a diagnosis of MDD and documented exposure to childhood neglect assessed with validated instruments.

Random-effects model based on the inverse variance method was applied to calculate pooled ORs and between-study heterogeneity was quantified using Cochran’s Q and I² statistics. Meta-regression models were performed to explore potential moderators influencing the outcomes across studies.

**Results:**

Twenty-two studies were included. Childhood neglect was significantly associated with MDD across all subtypes: unspecified neglect (OR = 4.14, 95% CI [1.73–9.89]; I² = 67.7%, p = 0.015), emotional neglect (OR = 3.71, 95% CI [2.33–5.88]; I² = 85.1%, p < 0.001), and physical neglect (OR = 3.30, 95% CI [2.22–4.91]; I² = 79.7%, p < 0.001). High heterogeneity was observed, particularly for emotional and physical neglect, reflecting variability across studies.

**Conclusions:**

These results are consistent with previous meta-analyses reporting strong associations between neglect and depression (Mandelli et al. Eur Psychiatry 2015; 30:665–680; Nelson et al. Br J Psychiatry 2017; 210:96–104; Norman et al. PLoS Med 2012; 9:e1001349), although the ORs in the present study were higher. Prior evidence indicates that neglect, especially emotional neglect, is more strongly linked to depressive outcomes than other forms of maltreatment (Humphreys et al. Child Abuse Negl 2020; 102:104361; Infurna et al. J Affect Disord 2016; 190:47–55).

The consistency and strength of these associations support the role of childhood neglect as a key vulnerability factor for MDD and underscore the importance of systematic screening and trauma-informed therapeutic strategies in clinical practice.

**Disclosure of Interest:**

None Declared

